# Ground and satellite based observation datasets for the Lower Mekong River Basin

**DOI:** 10.1016/j.dib.2018.11.038

**Published:** 2018-11-14

**Authors:** Ibrahim Nourein Mohammed, John D. Bolten, Raghavan Srinivasan, Chinaporn Meechaiya, Joseph P. Spruce, Venkat Lakshmi

**Affiliations:** a Science Applications International Corporation, Hydrological Sciences Laboratory, NASA Goddard Space Flight Center, Mail Code 617.0, Greenbelt, MD 20771, USA; b NASA Goddard Space Flight Center, Hydrological Sciences Laboratory, Mail Code 617.0, Greenbelt, MD 20771, USA; c Spatial Sciences Laboratory, Department of Ecosystem Science and Management, Texas A&M University, College Station, TX 77843, USA; d Asian Disaster Preparedness Center, SM Tower, 24th Floor, 979/69 Paholyothin Road, Samsen Nai Phayathai, Bangkok 10400, Thailand; e Science Systems and Applications, Inc., 10210 Greenbelt Rd # 600, Lanham, MD 20706, USA; f School of Earth Ocean and Environment, University of South Carolina, Columbia, SC 29208, USA

**Keywords:** Mekong river, Streamflow, Precipitation, Air temperature, Remote sensing, Soil characteristics

## Abstract

In ‘Satellite observations and modeling to understand the Lower Mekong River Basin streamflow variability’ [1] hydrological fluxes, meteorological variables, land cover land use maps, and soil characteristics and parameters data were compiled and processed for the Lower Mekong River Basin. In this work, daily streamflow time series data at nine gauges located at five different countries in the Mekong region (Thailand, Laos People׳s Democratic Republic (PDR), Myanmar, Cambodia, and Viet Nam) is presented. Satellite-based daily precipitation and air temperature (minimum & maximum) data is processed and provided over the entire basin as part of the dataset provided in this work. Moreover, land cover land use raster data that contains 18 classes that cover agriculture, urban, range and forests land cover land use classes for the basin is offered. In addition, a soil data that contains physical and chemical characteristics needed by physically based hydrological models to simulate the cycling of water and air is also provided.

**Specifications table**

**Table:** 

Subject area	Environmental Sciences
More specific subject area	Hydrology, Remote sensing
Type of data	Figure, Tables and grids
How data was acquired	Station measurement, remote sensing, soil maps and tables, and geographic information systems modeling
Data format	Raw data, analyzed
Experimental factors	Different observations were compiled and processed to produce maps and time series data of hydrology related inputs and fluxes, as well as climate for the Lower Mekong Basin
Experimental features	Maps of land cover and land use, and soil characteristics, as well as continuous time series data of streamflow, precipitation, and minimum and maximum air temperatures at the Lower Mekong Basin
Data source location	Thailand, Laos People׳s Democratic Republic (PDR), Myanmar, Cambodia, and Viet Nam, i.e., (north-south) from 22° 46′ 30′′ N to 11° 51′ 15′′ N, and (east-west) from 99° 1′ 17′′ E to 108° 46′ 22′′ E
Data accessibility	Data available within the article
Related research article	I. N. Mohammed, J. D. Bolten, R. Srinivasan, V. Lakshmi, Satellite observations and modeling to understand the Lower Mekong River Basin streamflow variability, J. Hydrol. 564 (2018) 559–573. doi:10.1016/j.jhydrol.2018.07.030[1]

**Value of the data**

• The satellite dataset benefits hydrologic modeling at poor spatial in-situ data regions such as the Lower Mekong River Basin.
• The dataset can assist to understand the water balance in the Lower Mekong River Basin.
• The dataset is essential in hydrological modeling in the Mekong region since it contains new developed land cover land use, and soil characteristics layers.

## 1 Data

**Fig. 1:**
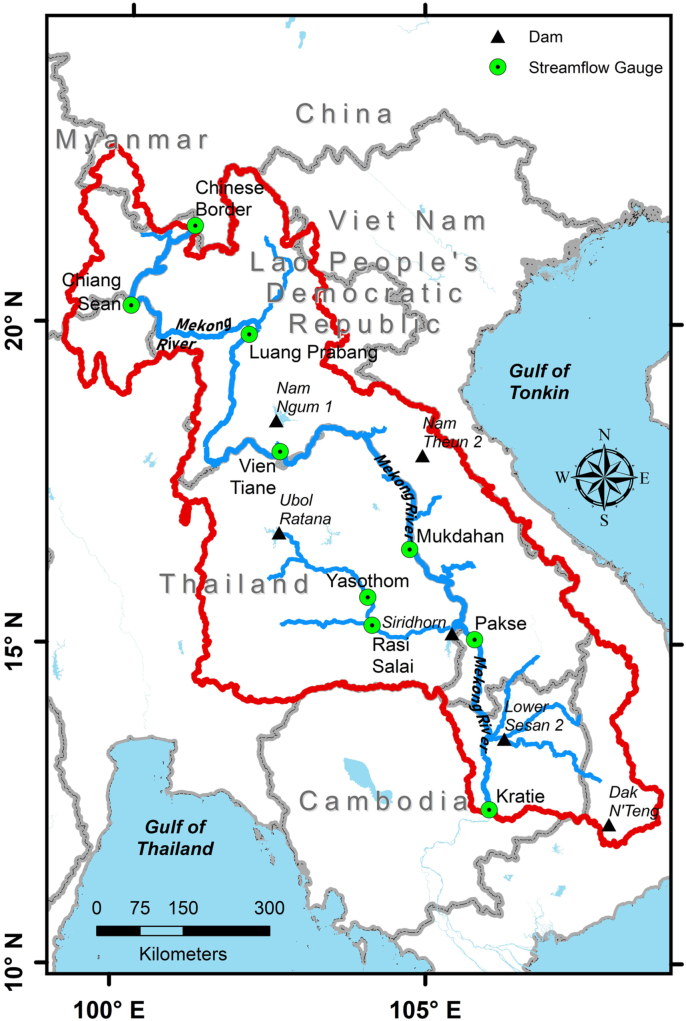
The Lower Mekong River Basin. The streamflow gauges depicted are described in Table 1. The dams shown are outlined in Table 2.

This paper reports various hydrological time series and remote sensing data that was used to model and understand the streamflow variability in the Lower Mekong River Basin (LMRB) [1]. Mohammed et al., [1] used the Soil & Water Assessment Tool (SWAT) hydrologic model (https://swat.tamu.edu/) to simulate hydrological fluxes in the LMRB and explore the streamflow regime changes as a result of expected upstream flow changes (i.e., the Chinese part of the Mekong River). Fig. 1 gives the layout of the Lower Mekong River Basin. The Lower Mekong River Basin begins when the Mekong River leaves the Chinese province of Yunnan and enters the Golden Triangle where the country borders of Thailand, Laos People׳s Democratic Republic (PDR), China and Myanmar come together. The Mekong River ends in a large delta before exiting to the South China Sea. The Lower Mekong River Basin referenced in this paper does not cover the Mekong River basin area south of Kratie, Cambodia (Fig. 1).

The LMRB streamflow data reported is observed and collected by various agencies in the Lower Mekong Region. The LMRB streamflow time series data that we present here was obtained from the Mekong River Commission (MRC) hydrological respiratory (http://www.mrcmekong.org/). Interpolation was carried out using a recent observed level data acquired from the Asian Preparedness Disaster Center (ADPC) geospatial and climate resilience teams (http://www.adpc.net/) to update and fill the gaps of the streamflow time series data we present. The dam data we report here was obtained from the Greater Mekong Consultative Group for International Agricultural Research (CGIAR) Program on Water, Land and Ecosystems [2].

The precipitation data we report here was obtained from the Tropical Rainfall Measurement Mission (TRMM) [3], and combined with the Integrated Multi-satellite Retrieval for the Global Precipitation Measurement mission (IMERG) [4] remote sensing data products. The TRMM, and GPM remote sensing data products can be accessed at https://pmm.nasa.gov/data-access/downloads/. The TRMM dataset we report here was processed from a daily 0.25 × 0.25° accumulated precipitation that is generated from the near real-time 3-hourly (TMPA /3B42RT) product. We also report precipitation data obtained from the IMERG dataset. The IMERG dataset presented here is the Global Precipitation Mission (GPM) Level 3 IMERG *Final* Daily 0.1 × 0.1 degree (GPM_3IMERGDF) data product, which is derived from the half-hourly data product (GPM_3IMERGHH). The derived result represents the final estimate of the daily accumulated precipitation in millimeters.

Minimum and maximum daily air temperature data we describe here was calculated from air temperature record retrieved from the Global Land Data Assimilation System (GLDAS) simulation data products [5]. The goal of the GLDAS [5] is to ingest satellite and ground-based observational data products, using advanced land surface modeling and data assimilation techniques, in order to generate optimal fields of land surface states and fluxes. For this paper, we used the GLDAS Noah Land Surface Model L4 3-h 0.25 × 0.25° (GLDAS_NOAH025_3H.2.1) data product available at https://disc.gsfc.nasa.gov/.

The land cover land use data we present was produced from multiple 2010 land cover land use maps at a spatial resolution of a 0.25 km for the Lower Mekong Basin [6]. The land cover land use maps produced and presented herein used the Moderate Resolution Imaging Spectroradiometer (MODIS) monthly Normalized Difference Vegetation Index (NDVI) data and circa 2010 dry season Landsat reflectance data as the primary data sources as well as high resolution satellite data from Google Map/Earth, and other reference data from the MRC. The spatial scale of the land cover land use data presented here is 90 m.

The soil data we report here was processed from the Harmonized World Soil Database (HWSD) [7]. The HWSD data was obtained from the Food and Agriculture Organization of the United Nations (FAO) and processed to be compatible with the SWAT hydrological model. The HWSD soil data is a 30 arc-second raster database with over 15,000 different soil mapping units that combines existing regional and national updates of soil information worldwide.

## 2 Experimental design, materials, and methods

The streamflow time series data is presented with this paper as a spreadsheet in Appendix A.1. The streamflow gauge names, gauge identification codes, and record period for each gauge is shown in Table 1.

**Table 1:** The Lower Mekong River streamflow data. Discharge units are in m^3^/s. Temporal scale is daily.

**Station name**	**Code**	**Country**	**Start date**	**End date**
Chinese Border	010000	China	1/1/1985	12/31/2007
Chiang Sean	010501	Thailand	1/1/1960	12/31/2016
Luang Prabang	011201	Laos, PDR	1/1/1939	12/31/2016
Vientiane	011901	Laos, PDR	1/1/1913	12/31/2016
Mukdahan	013402	Thailand	1/1/1923	12/31/2016
Pakse	013901	Laos, PDR	1/1/1923	12/31/2016
Kratie	014901	Cambodia	1/1/1924	12/31/2016
Yasothom	370104	Thailand	1/1/1952	12/31/2003
Rasi Salai	380134	Thailand	1/1/1979	12/31/2003

Dams within the Lower Mekong River Basin that are either already commissioned or still under construction and have a maximum reservoir area greater than or equal to 280 km^2^ are reported in this paper. Table 2 gives dam data at the LMRB that was used in simulating streamflow [1].

**Table 2:** Dams data within the Mekong Basin obtained from the CGIAR [2]. The COD column refers to the Commercial Operation Date (i.e. when the dam was commissioned).

**Name**	**Country**	**River**	**Latitude**	**Longitude**	**Function**	**Status**	**COD**	**Installed capacity**	**Mean annual energy**	**Height**	**Crest length**	**Full supply level**
							**Year**	**Megawatts**	**Gigawatts**	**meter**	**meter**	**Million m**^3^
Lower Sesan 2	Cambodia	Se San	13° 33' 5"	106° 15' 50"	Hydropower	Under construction	2019	480	2,311.8	45	7,729	1,790
Nam Ngum 1	Laos, PDR	Nam Ngum	18° 31' 52"	102° 32' 51"	Hydropower	Commissioned	1971	149	1,006.00	75	468	4,700
Nam Theun 2	Laos, PDR	Nam Theun	17° 59' 50"	104° 57' 8"	Hydropower	Commissioned	2009	1075	5,936.00	48	325	3,500
Siridhorn	Thailand	Lam Dom Noi	15° 12' 23"	105° 25' 45"	Hydropower	Commissioned	1971	36	86.00	42	940	1,967
Ubol Ratana	Thailand	Nam Pong	16° 46' 31"	102° 37' 6"	Hydropower	Commissioned	1966	25.2	57.00	35.1	885	2,559
Dak N'Teng	Viet Nam	Dak N'Teng	12° 11' 46"	107° 55' 36"	Hydropower	Commissioned	2011	13	52.80	31	315	25.49

**Max reservoir area**	**Est. cost**	**Power destination (%)**							**Developers**	**Owner/ operator**	**Notes**
**km**^2^	**Mil. US$**	**LAO**	**THA**	**CAM**	**VN**	**CHN**	**MYN**	**IND**			
335	781.52	0	0	30	70	0	0	0	HydroLancang and Royal Group		Location derived from Sesan, Sre Pok, and Sekong (3Ss) River Basins Development Study in Kingdom of Cambodia, Lao People’s Democratic Republic, and Socialist Republic of Viet Nam. ADB - RETA 40082.
370	97	80	20	0	0	0	0	0		EdL Gen (100%)	
450	1300	7	93	0	0	0	0	0		Nam Theun Power Co. (EDF: 40%; EGCO (Thailand): 35%; GoL: 25%)	
288		0	100	0	0	0	0	0			
410		0	100	0	0	0	0	0	Electricity Generating Authority of Thailand	Electricity Generating Authority of Thailand	https://en.wikipedia.org/wiki/Ubol_Ratana_Dam
323		n/a	n/a	n/a	n/a	n/a	n/a	n/a			Data provided by IWRP

The precipitation data for the whole LMRB is presented with this paper as a spreadsheet named ‘Precipitation’ in Appendix A.2 which gives climate data for the study area. The precipitation data units are in millimeters. The temporal span for the data is from 1 January 2001 to 31 December 2015. Area weighted average methodology was used to obtain an aggregated precipitation time series data for the LMRB. Since IMERG data products are only available from 12 March 2014 to present, we used the TRMM rainfall data (3B42RT) for time periods earlier than 12 March 2014. A nearest neighbor methodology was used to fill the IMERG data points with the TRMM data points as an approximation during the 1 January 2000 to 11 March 2014 time period [8]. Since TRMM and IMERG data do not have the same spatial resolution (i.e., 0.25 and 0.1 degree respectively), a methodology was presented in Mohammed et al. [8] to address the spatial scale differences.

The air temperature data for the whole LMRB is presented with this paper as spreadsheets named ‘Tmin’ and ‘Tmax’ in Appendix A.2. The air temperature data (minimum and maximum) units are in degree Celsius. We calculated the daily minimum and maximum temperatures by finding the minimum and maximum air temperatures for each day at each grid within the study watershed by searching for minima and maxima over the three hours air temperature data values available for each day and grid. Area weighted average methodology was used to obtain an aggregated air temperature (min/max) time series data for the LMRB.

The MODIS monthly NDVI images used to produce the land cover land use map presented (Appendix A.3) were derived from MOD09 and MYD09 8-day reflectance data that was temporally processed using the Time Series Product Tool custom software package [9]. The land cover land use map produced were developed primarily from unsupervised classification of the 2010 MODIS NDVI data, including several agricultural and forest types. The Landsat data was used with a combination of unsupervised and supervised classification methods to map land cover land use classes that were regionally scarce but locally common, including bamboo forest scrub, industrial forest plantation, urban, and water classes. Geographic Information System techniques were then applied to integrate the MODIS and Landsat classifications into singular land cover land use map for entire LMRB. The land cover land use classes presented here for the LMRB are listed in Table 3. In general, the land cover land use classes can be categorized into agricultural land classes, forest type classes, grass lands, urban lands, and water. Appendix A.3 gives the land cover land use raster grid for the LMRB along with the raster projection information.

**Table 3:** Land cover land use classifications. Raster value refers to the land cover land use ascii raster file provided in Appendix A.3.

**Raster value**	**Name**
10	Water
15	Barren - rock outcrops
16	Urban
21	Agriculture - rice - 1 crop per year
22	Agriculture - rice - 2 crops per year
23	Agriculture - mixed annual crops - other than rice
24	Agriculture - shifting cultivation - cleared before 2010 - herbaceous cover
25	Agriculture - shifting cultivation - cleared in 2010
26	Agriculture - shifting cultivation - partially cleared in 2010
31	Deciduous shrubland - mixed scrub/herbaceous/low broadleaved forest
32	Forest/scrub - deciduous broadleaved - low height
33	Forest - deciduous/evergreen - low/medium height
34	Forest - evergreen broadleaved - medium/tall height
35	Forest - evergreen/deciduous broadleaved - low/medium height
36	Bamboo scrub/forest - low height - mostly evergreen
41	Grassland - sparse vegetation
42	Industrial forest plantation - low/medium height
43	Wetland - mixed shrubland/herbaceous riparian areas

The soil data reported here was produced to meet the hydrological modeling needs in Mohammed et al., [1]. The soil database development was intended to be as an input for the SWAT model development at the Lower Mekong. However, the methodology and parameters presented here can aid other studies. The saturated hydraulic conductivity, a quantitative measure of a saturated soil׳s ability to transmit water when subjected to a hydraulic gradient, reported here as “SOL_K(layer#)” has been estimated using the Soil Water Characteristics and the SPAW Hydrology and Water Budgeting software [10]. Table 4. gives a summary of the various soil parameters presented in the Appendix A.4 soil table ‘LMRB_usersoil.xlsx’. Appendix A.4 also gives the soil raster grid for the LMRB using the same projection highlighted earlier in the land cover land use raster grid (Appendix A.3).

**Table 4:** Soil parameters for the soil database presented in Appendix A.4.

**No**	**CODE**	**Definition**	**Note**
1	MUID	The harmonized soil mapping unit identifier of HWSD providing the link to the GIS raster	Available from HWSD
2	SNAM	Soil Name	Shown as a concatenate between Texture and MUID
3	NLAYERS	Number of available layers	Available from HWSD
4	HYDRGRP	Soil hydrologic group (A, B, C, or D)	Calculated using guidelines from Table 7.1, Chapter 7, Hydrologic Soil Groups, The National Engineering Handbook (NEH) Part 630 Hydrology (https://directives.sc.egov.usda.gov/OpenNonWebContent.aspx?content=17757.wba)
5	SOL_ZMX	Maximum rooting depth of soil profile (mm)	calculated from SOL_Z(layer#)
6	TEXTURE	Texture of soil layer	Shown here as texture1-texture2-… to reflect the number of layers
7	SOL_Z(layer #)	Depth from soil surface to bottom of layer (mm)	Available from HWSD
8	SOL_BD(layer #)	Moist bulk density (Mg/m^3^ or g/cm^3^)	Available from HWSD
9	SOL_AWC(layer #)	Available water capacity of the soil layer (mm H_2_O/mm soil)	Available from HWSD
10	SOL_K(layer #)	Saturated hydraulic conductivity (mm/hr)	Calculated using sand, clay, organic matter, and soil moisture percentages. The Soil Water Characteristics software developed by USDA Agricultural Research Service in cooperation with the Department of Biological Systems Engineering, Washington State University, has been used to estimate saturated hydraulic conductivity values. Results obtained for the different soil texture in this database are: CLAY (HEAVY) 0.78 mm/hr; CLAY (LIGHT) 2.06 mm/hr; CLAY LOAM 4.56 mm/hr; LOAM 18.6 mm/hr; LOAMY SAND 91.26 mm/hr; SAND 114.05 mm/hr; SANDY CLAY 0.84 mm/hr; SANDY CLAY LOAM 7.84 mm/hr; SANDY LOAM 50.34 mm/hr; SILT 18.95 mm/hr; SILTY CLAY 3.81 mm/hr; SILTY CLAY LOAM 5.93 mm/hr; SILT LOAM 12.19 mm/hr.
11	SOL_CBN(layer #)	Organic carbon content (% soil weight)	Estimated using Organic Matter(%) divided by 1.724
12	CLAY(layer #)	Clay content (% soil weight)	Available from HWSD
13	SILT(layer #)	Silt content (% soil weight)	Available from HWSD
14	SAND(layer #)	Sand content (% soil weight)	Available from HWSD
15	ROCK(layer #)	Rock fragment content (% total weight)	Available from HWSD
16	SOL_ALB(layer #)	Moist soil albedo	Calculated using Equation [15] in the USDA Water Erosion Prediction Project, 1985, Baseline Soil Erodibility Parameter Estimation. Baumer, O.W. 1986. Estimation of hydraulic parameters from known soil properties. Transaction of the ASAE Conference, St. Luis Obispo, CA. 29 June-2 July 1986. Baumer, O.W. 1990. Prediction of soil hydraulic parameters. In: WEPP Data Files for Indiana. SCS National Soil Survey Laboratory, Lincoln, NE. (http://milford.nserl.purdue.edu/weppdocs/usersummary/BaselineSoilErodibilityParameterEstimation.html)
17	USLE_K(layer #)	USLE equation soil erodibility (K) factor	Calculated using guidelines in SWAT handbook, Chapter 22 (https://swat.tamu.edu/media/69365/ch22_input_sol.pdf)

## Appendix A Supplementary materials

The following materials contain the Keyhole Markup (KMZ) Google maps file of the most important data features described in this article. Other materials are comprised of spreadsheet and grid files as discussed in this article.

Map Land cover and land use raster data in spatial reference of the European Petroleum Survey Group (ESPG: 32648).

Map: Keyhole Markup file (KMZ) contains the Google maps

.

Soil raster data in spatial reference of ESPG: 32648. Soil characteristics table is also included.

.

Daily aggregated precipitation and air temperature time series data.

.

Daily streamflow time series data.

## Footnotes

Appendix A Supplementary data associated with this article can be found in the online version at https://doi.org/10.1016/j.dib.2018.11.038. The following materials contain the Keyhole Markup (KMZ) Google maps file of the most important data features described in this article. Other materials are comprised of spreadsheet and grid files as discussed in this article.
